# Strategic Sexual Signals: Women's Display versus Avoidance of the Color Red Depends on the Attractiveness of an Anticipated Interaction Partner

**DOI:** 10.1371/journal.pone.0148501

**Published:** 2016-03-09

**Authors:** Daniela Niesta Kayser, Maria Agthe, Jon K. Maner

**Affiliations:** 1 Department of Psychology, University of Potsdam, Potsdam, Germany; 2 Department of Psychology, Ludwig-Maximilians-University of Munich, Munich, Germany; 3 Department of Management and Organizations, Kellogg School of Management, Northwestern University, Evanston, IL, United States of America; IRCCS E. Medea, ITALY

## Abstract

The color red has special meaning in mating-relevant contexts. Wearing red can enhance perceptions of women’s attractiveness and desirability as a potential romantic partner. Building on recent findings, the present study examined whether women’s (N = 74) choice to display the color red is influenced by the attractiveness of an expected opposite-sex interaction partner. Results indicated that female participants who expected to interact with an attractive man displayed red (on clothing, accessories, and/or makeup) more often than a baseline consisting of women in a natural environment with no induced expectation. In contrast, when women expected to interact with an unattractive man, they eschewed red, displaying it less often than in the baseline condition. Findings are discussed with respect to evolutionary and cultural perspectives on mate evaluation and selection.

## Introduction

No color draws as much attention as red: It can imply a warning (e.g., in the form of street signs or traffic lights), seduce people to buy products (e.g., in the form of advertisements, e.g. [1]), or attract potential romantic partners (e.g., through the display of red-colored clothing or make-up). The color red seems to be of particular importance in mating contexts. Several non-human primate species display red sexual swellings during peak fertility, for example, and those displays attract potential mates (e.g., [2]). This link between red and sexual attractiveness may also extend to humans: when interested in casual sex (but not other types of relationships), women were more inclined to wear red on a dating website [3].

Numerous studies in the U.S., Europe, and Africa suggest that men perceive female targets as more attractive and sexually desirable if they are wearing red clothing, if they are seen in front of a red background, or if they are pictured near a red product [4–8]. These effects of the color red are specific to opposite-sex (not same-sex) ratings of attractiveness and desirability, and do not influence ratings of the female targets’ other qualities, such as her overall likeability, kindness, or intelligence [4,6,9]. Even just carrying red-colored products (like a red laptop in comparison to a different-colored laptop) specifically enhanced female sexual attractiveness (but not perceived health or assertiveness) and only in the perception of men (but not women), thus substantiating the presumed red-attraction link [6]. Moreover, the color red enhanced men’s ratings of female attractiveness specifically for attractive (but not unattractive) faces [10]. Thus, findings suggest a highly specific link between the color red and female attractiveness in the domain of mating.

One explanation for the link between red, attractiveness, and desirability refers to socialization and learning processes. For instance, red roses, red hearts on Valentine’s Day, red lipstick, and red light districts promote an association between the color red on one hand, and love, passion, and romantic contexts on the other hand. Evidence suggests that red’s positive effect on attractiveness may be a result of its specific link to perceived sexual receptivity and desirability [11,7,12,8]. There may be deeper biological reasons for red’s association with sexuality, as well. For example, reddish skin tone can signal high levels of cardiovascular health [13] and there are important reproductive reasons for preferring potential mates who are healthy [14,15]. Indeed, several findings suggest that the link between red and sexuality may not be a product of societal conditioning alone, but might also have roots in humans’ biological heritage.

Several findings hint at the possibility that the association between red and sexuality is rooted in human evolutionary history. First, red ornamentation is associated with fertility and attraction in many nonhuman primates [2]. Second, there is cross-cultural evidence for the red-sexuality link. For instance, even though red has a generally negative connotation in traditional small-scale societies in rural Burkina Faso (representing bad luck, sickness, and death), the red-attraction link (specifically, the culturally appropriate expression of romantic attraction) is still present, as red enhances women’s attractiveness to men in that culture [16]. Similarly, numerous ethnographic records (e.g., the use of red ochre for grooming and wedding rituals in Africa [17], point to the possible universality of the link between red and sexuality. Third, the color red enhances men’s perceptions of young (but not older) women’s sexual attractiveness; that is, the effect is seen only in fertile women, not women in menopause or post-fertility, consistent with the possibility that red is connected to perceptions of a woman’s underlying reproductive value [10]. This body of research is consistent with the possibility that the link between red and attraction is rooted in human evolutionary biology. Cultural conditioning could, in turn, reinforce and extend this link.

Women, in particular, are viewed as more attractive by men when those women display the color red. For instance, men asked a target woman in red (compared with one not displaying red) more intimate questions and chose to sit closer to her [18]. Women wearing red clothing while working in a restaurant received better tips [19], and men were more likely to offer a red-dressed woman a car ride [11,20,21].

The link between red and female attractiveness may have implications for behaviors women use to attract highly desirable mates. For instance, women are more likely to wear red (and pink) when they are in the fertile phase (as compared to the non-fertile phase) of their menstrual cycle [22], a time during which women are also especially motivated to attract highly desirable sexual partners–those high in dominance, sexual attractiveness, and who display signs of high genetic quality [17,23].

In two recent studies, female participants who expected to talk to an attractive (relative to an unattractive) man were more likely to choose red clothing during the experimental session [24], providing the first experimental evidence that women use the color red strategically to attract highly desirable (as compared with less desirable) mates. However, the strength of this evidence was limited by the fact that women arrived at the study and, after being instructed that they would meet with an opposite sex interaction partner, were explicitly asked to choose a prototypical (“fire-engine”) red (versus control color) shirt to wear as part of the experiment. The study was limited by the fact that this specific, obvious type of choice might have appeared rather artificial for the participants. That is, due to the highly explicit nature of the choice, this approach may have introduced participant demand characteristics and elicited a level of conscious decision making that goes beyond the presumably more implicit choices that typically guide women’s display of color. Thus, the approach taken in those studies may have fallen short of tapping into the subtle color display behaviors women use to attract men. In addition, the striking level of red used in these prior studies (i.e., “fire-engine” red) does not sufficiently examine color choice, because other reddish shades which are less eye-catching might perhaps be chosen by women who are (consciously or unconsciously) interested in mate search, but who wish to avoid standing out too much and would thus avoid prototypical red. Although those previous studies provide a useful start, a more definitive test is needed.

In the current investigation, women expected to interact with an attractive (or unattractive) male research assistant and arrived at the experiment displaying whatever clothing or other accessories they chose to display that day. No special instructions pertaining to color, attire, etc. were used. Thus, women’s color displays (in clothing, accessories, or make-up) were entirely natural. This approach eliminated the potential for participant demand, reflected a high level of ecological validity, and reflected the presumably implicit processes through which women choose to display the color red.

As noted earlier, women’s display of the color red should be used strategically and selectively to attract high quality mates. That is, women should not send signals of interest indiscriminately to all men. Women tend to be highly selective in the context of mate selection [25], and this high level of choosiness would be expected in the use of strategic color displays. Good genes theories of sexual selection, for instance, suggest that attractiveness can signal high genetic quality [26–29] and preferences for attractive individuals evolved because those preferences enhanced reproductive success [30,31]. In contrast, women tend to actively avoid men who lack desirable mating-related traits, because mating with a man who lacks desirable traits carries the risk of investing in offspring who will not inherit high levels of reproductive fitness [32].

Indeed, while women would be expected to display red in order to attract a highly desirable man, they would also be expected to avoid displaying the color red when expecting to interact with an undesirable man. No previous evidence speaks directly to whether women might avoid displays of red when confronted with men who lack desirable adaptive traits [33, 34]. Thus, in the current investigation, we predicted that, relative to a baseline measure, women expecting to interact with a relatively unattractive man would avoid displaying the color red. Conversely, we also expected to replicate and extend previous evidence [16] for heightened displays of red when women expected to interact with an attractive man.

## Materials and Methods

### Ethics Statement

This study was approved by the Ethics Committee of the Ludwig-Maximilians-University (No DFG Ni/1115/2-1) in Munich in accordance with the ethical standards expressed in the Declaration of Helsinki. All participants gave verbal informed consent and were thoroughly debriefed. The individuals’ verbal consent was obtained at two separate points: first it was obtained after individuals responded to the contact email by affirming that they wanted to participate (this affirmative expression was qualified written consent). The second consent was obtained before and after the participants had their photograph taken. They were thoroughly informed that they did not need to participate in this additional procedure and assured that they could discontinue and leave the experiment at any point of time. When the experimenter asked for the participant’s consent, he emphasized that they would receive their credit even if they decided not to participate in this study. Verbal consent was considered to be sufficient, because it was ensured that data were stored and analyzed anonymously. We also considered verbal consent to be more appropriate (compared with written consent): Obtaining verbal consent guaranteed the participants that neither their name nor their signature was associated with their picture in any way, hence guaranteeing the participants’ anonymity. Additionally, both before and after taking the picture the participants were reassured that they could discontinue the study at any point. No one withdrew consent to having their picture used in this study. The photographs are used solely for data analysis and are not published. The procedure of verbal informed consent including the consent protocol was approved by the Ethics Committee based on this information.

### Participants

Seventy-nine women participated in the experiment in return for a modest cash payment (5 Euros). The mean age of participants was 22.4 years (range = 18–43). Data analyses were restricted to self-reported heterosexual and bisexual individuals. Five participants who reported that they were lesbian were excluded from the analyses, because the design of the study did not provide an appropriate test of their mate selection. This research has been conducted and approved by the authors' Institutional Review Board (IRB) at the Ludwig-Maximilians-University in Munich, Germany, according to the principles expressed in the Declaration of Helsinki.

### Design and Procedure

If consent was given to be contacted for the study, participants received a personal confirmation via e-mail (see: ethics statement). The confirmation e-mail contained information about where and when the study would take place, as well as a photograph depicting a male research assistant who allegedly was to conduct the study. The two photographs were selected from a larger US based college picture contingent on their attractiveness means and standard deviations while also assuring that targets and respondents did not know each other. Moreover, respondents and stimulus persons were all of the same ethnicity (European-Caucasian) and same age category (i.e., young adults in their twenties). The attractiveness of the research assistant was manipulated by including a picture in the email, ostensibly provided to make it easier for the participants to recognize the person in charge of the study.

The attractiveness of the two photographs was pre-rated by an independent sample of 20 female students. Two items were used: “How attractive is this person?” (1 = not at all attractive, 9 = very attractive) and “How handsome is this person?”(1 = not handsome at all, 9 = very handsome). The attractive male photo was rated as above the midpoint in attractiveness (*M* = 6.63, *SD* = 0.89; *t*(19) = 8.20, *p* < .001), whereas the relatively unattractive male photo was rated as below the midpoint (*M* = 3.90, *SD* = 1.35, *t*(19) = -3.63, *p* < .01). A follow-up ANOVA showed that the attractiveness ratings of the two men were not significantly different from each other in their deviation from the midpoint, F(1, 19) = 1.84, *p* = .190, making these two stimuli ideal for purposes of comparison.

Upon arriving for the session, participants were informed that the research assistant depicted in the email was unavailable, so the session would be conducted by a different experimenter. Following informed verbal consent (see: ethics statement), participants were offered 5 Euro by a trained research experimenter (in addition to their base payment for participation) if they agreed their photo to be taken (only one woman declined to have her photo taken) followed by a standardized questionnaire.

The experimenter took two photos, one close-up of the woman’s face and the second of her entire body, to ensure that all aspects of her clothing, accessories, and makeup were visible in the photos. After the photos were taken, participants answered questions pertaining to general demographics, their relationship status (based on an item that provided five relationship options) and their self-perceived attractiveness (“how attractive do you think other-sex individuals perceive you?”).

Participants then underwent a suspicion probe and debriefing. Three out of 74 students indicated that they had suspected that the research assistant’s photo was relevant for the study. The pattern of results was identical whether or not those three participants were retained in the analyses; thus, the results reported below refer to the analysis of all 74 participants.

We also sought to compare the two experimental conditions to a naturalistic baseline frequency of displaying red within this population. For this purpose, two independent raters observed a random sample of 200 women in the largest student cafeteria at the university and coded whether or not each woman displayed the color red. These data were collected during the same semester as the experiment. Coders were blind to the hypotheses of the study and the results of the main experiment. Among the 200 women observed, 64 (32%) displayed some form of red, whereas 136 (68%) did not display any red.

Regarding the coding procedure, we referred to commonly used counting procedures in the literature [22].

The range of reddish hues included pink, red and scarlet, thereby including prototypical shades of red, but excluding atypical shades of red, such as orange, maroon or purple.

Our primary analysis tested whether women in the attractive experimenter condition relative to those in the unattractive experimenter condition were more likely to display red in their attire, accessories, and make-up. Two individuals, blind to the hypothesis and experimental condition, coded use of the color red in participants’ clothes, accessories, and make-up. In coding the color of the participant photographs, the independent raters used the same, calibrated monitor/display in the research lab. We kept the conditions in which the photos were taken constant (e.g., the amount of the light influence was equivalent; the location was always in front of the same off-white wall in the lab). Female participants were categorized as displaying red if they showed red on any part of their clothing (e.g., shirt or dress), accessories (e.g., bag or scarf) or make-up (e.g., lipstick). Inter-coder agreement was good (Kappa > .90 *p* < .001); a third rater resolved discrepancies.

## Results

We used logistic (binary) regression with attractiveness condition as the predictor variable (attractive = 1, unattractive = 0) and display of red as the dependent variable (red displayed = 1, no red displayed = 0). Results confirmed that women who expected to interact with an attractive experimenter were substantially more likely to display red than those who expected to interact with an unattractive experimenter, B = 1.91, Wald χ^2^ = 11.86, *p* < .001, χ^2^(1) = 13.69, *p* < .001, Nagelkerke R^2^ = .231, Odds ratio = 6.78. Among the 27 women displaying red, twenty-one (77.80%) were in the attractive condition compared to six women (22.2%) in the unattractive condition; among the 47 women not displaying red, 16 (34%) were in the attractive condition and 31 (66%) were in the unattractive condition (see Table 1). These findings are consistent with the hypothesis that women who expected an attractive experimenter would be more likely to display red than those who expected an unattractive experimenter.

**Table 1:** Red display of women in the unattractive and attractive experimenter condition.

Stimulus attractiveness	Red not displayed	Red displayed	Total
Attractive	16	21*	37
	34%	77.80%	50%
Unattractive	31	6*	37
	66%	22.20%	50%
Total	47	27	
	100%	100%	

Note.

*Between columns percentages differ at a p < .05 level.

To include a more precise (i.e., interval) measure, two independent raters (both blind to hypotheses and experimental condition) estimated the amount of the color red in the women’s attire by indicating on a 6-point-Likert-Scale (from 0 = not at all to 5 = very much) (1) *how much* and (2) *how obviously* the color red was displayed by participants (inter-rater reliability was high: α = .91). We performed a t-test to compare the two scores for the attractive versus the unattractive condition. Analyzes revealed that (1) the *quantity* of displaying red was significantly greater in the attractive (*M* = 1.09, *SD* = 1.30; range = 0–4) compared with the unattractive (*M* = .42, *SD* = 1.06; range = 0–4) condition, *t*(73) = 2.45, *p* = .017, Cohen’s *d* = 0.57; (2) likewise, the *perceived conspicuousness* of displaying red was significantly higher in the attractive (*M* = 1.23, *SD* = 1.46; range = 0–4) as compared with the unattractive (*M* = .49, *SD* = 1.32; range = 0–4) condition, *t*(73) = 2.46, *p* = .023, Cohen’s *d* = 0.59.

We also examined whether women in the two experimental conditions differed from those assessed under naturalistic baseline circumstances (i.e., the random sample of students observed on campus; for reference, please see S1 Fig). For the *attractive* condition (56.8% display of red), the confidence interval around the frequency of red displays ranged from 41.19% to 71.66%, which was significantly different from baseline (32%), *t*(73) = 4.28, *p* < .001 (two-tailed). For the *unattractive* condition (16.2% display of red), the confidence interval ranged from 5.14% to 27.25%, which was also significantly different from the baseline (32%), *t*(73) = 3.67, *p* < .001 (two-tailed). Thus, whereas women expecting an attractive interaction partner displayed the color red more often than would be expected by chance, women expecting an unattractive interaction partner displayed the color less often than would be expected by chance.

In our sample, all results remained the same when including age (age range = 18–43), relationship status, and self-perceived attractiveness as covariates. However, it is possible that replications with larger participant samples and higher statistical power might reveal such factors to be influential.

## Discussion

Do women strategically display the color red when anticipating an interaction with an attractive man? And do they actually avoid wearing red when anticipating an interaction with a relatively unattractive man? Results from the current study suggest that the answer to both questions is: yes. Consistent with prior research on the link between red and sexuality, our findings indicate that women’s use of red clothing, accessories, and/or make-up can indeed serve as a subtle and strategic indicator of sexual interest. A higher percentage of female participants displayed red when they expected to interact with an attractive (vs. an unattractive) male experimenter. Moreover, the percentage of participants wearing red in the attractive condition was higher than in a naturalistic baseline condition, and–notably–the percentage of women wearing red in the unattractiveness condition was lower than in the naturalistic baseline.

The current study overcomes limitations of prior research by measuring women’s natural behavior (i.e., spontaneous choice of clothing, accessories, and make-up). Women actually selected their outfit from their *own* clothes and attire in their natural environment (i.e., at their own home) and were not alerted to the study’s aim of assessing their choice of color.

From an evolutionary perspective, the quality of a romantic partner is especially important for women, because women’s potential to produce offspring is more limited than men’s, and because women’s level of initial obligatory parental investment is higher than it is for men (see [35]). Accordingly, women should be particularly thoughtful about finding a partner with high mate value *and* also careful to avoid a partner with low mate value. Thus, catching the attention of unattractive men might be maladaptive, consistent with theories emphasizing the importance of avoiding unattractive mates for women [33,34]. Indeed, several evolutionary theories suggest that above and beyond seeking attractive mates, people (and women in particular) are motivated to avoid interacting with or mating with unattractive individuals (e.g., [36,37]. Taken together, women who display mating signals to physically *unattractive* men might risk lowering their reproductive success. Thus, our results are consistent with the idea that the use of red in clothing, accessories, and/or make-up reflects an adaptive strategy to enhance one’s chances to attract (vs. avoid) a partner with high (vs. low) mate value.

Given that clothing choices are an essential part of people’s daily life, it is interesting to consider the implications of apparel decisions more broadly (see [38]). In general, women might avoid red in situations in which their aim is to blend in rather than to stand out or in situations in which they wish to avoid unwanted mating attention. These results might not only have relevance for designers and clients of online dating services who are interested in an “optimal” appearance, but also for people working in marketing and in the field of communication (i.e. for using the color red to persuade people to buy certain products or for conveying specific messages, such as in political or societal contexts). In addition, it is possible that the use and avoidance of the color red might have implications in clinical contexts: In particular, women who are socially anxious or high in introversion might be cautious in their use of red. The use of red could also have implications for workplace interactions, as the color red could signal (potentially inappropriate) levels of attraction toward opposite-sex coworkers. The degree to which the present results and these broader implications also apply to men (i.e., whether they also strategically use the color red in mating contexts; for instance, to signal attractiveness, status or dominance) is an open question worthy of subsequent research (see [39,8].

Future research might continue to examine moderators of the link between the color red and displays of sexual interest aimed at attracting highly desirable male partners. For example, women in the fertile window of their menstrual cycle are especially interested in mating with highly attractive men, while also avoiding less attractive men [23]. Importantly, most recent research found that women were more likely to wear red during the fertile window than on other cycle days [40]. Consequently and based on this evidence, it would be interesting to test whether women display the color red strategically during the most fertile time of their cycle to attract men possessing desirable traits, while also downplaying the display of red to avoid unwanted attention from men who lack desirable traits (cf. [41].

Moreover, weather and season may moderate the use of red in sexual signaling, as women may use red displays more in the winter, when other types of signaling (e.g., wearing minimal clothing) are less likely [42]. It is interesting to note that relationship status did not moderate the pattern of effects observed in this research, implying that women in a relationship still displayed red when expecting to meet the attractive experimenter. Whether this null effect of relationship status would generalize to other samples with higher statistical power or to a different population of participants, such as an older, more committed sample (e.g., married women), provides interesting questions for further investigation. Forms of sexual signaling can include both conscious and nonconscious processes. Thus, another interesting research question pertains to whether or not (or to what extent) women’s choice of the color red reflects a conscious versus implicit sexual attraction strategy.

Another interesting question pertains to whether women might wear the color red as a way of competing with other women. Women often enhance their attractiveness as a way of competing with other women to gain the attention of desirable men [43]. This phenomenon may extend to color displays, as the color red appears to be one feature that enhances perceptions of a woman’s attractiveness. Future research would profit from assessing whether intrasexual competition leads women to heighten their display of the color red.

Moreover, the value of our baseline condition was limited by the fact that participants were evaluated under conditions that differed from those under which our focal participants were evaluated. It is possible that participants may have dressed when going to the cafeteria than when showing up to an experiment. Thus, although our baseline sample was randomly selected and was from the same population as our participants, it would be important for future studies to include a more rigorous control condition (e.g., by using the same confirmation letter, just without including a picture of the alleged experimenter). Future research would also benefit from including a larger participant sample based on an a-priori power analysis.

## Conclusion

In conclusion, the present research adds to an emerging body of work showing that red carries special meaning in mating-related contexts. Women appear to selectively use or avoid using red as a sexual signal in their choice of clothing, accessories, and makeup. This research reveals a subtle but potentially powerful sexual strategy that women use to attract highly desirable mates while potentially avoiding unattractive ones.

## Supporting Information

S1 Fig Women’s display of red as a function of the male experimenter’s attractiveness. (PDF)

S1 Table Red display of women in the unattractive and attractive experimenter condition. (PDF)
