# Supplementary material for: Strategic Sexual Signals: Women's Display versus Avoidance of the Color Red Depends on the Attractiveness of an Anticipated Interaction Partner
Source: PLoS One. 2016 Mar 9;11(3):e0148501. doi: 10.1371/journal.pone.0148501 (PMC4784733; doi:10.1371/journal.pone.0148501)
Supplement: S1 Fig — (PDF) [file pone.0148501.s001.pdf]

Figure 1. Displaying red as a function of experimenter's attractiveness

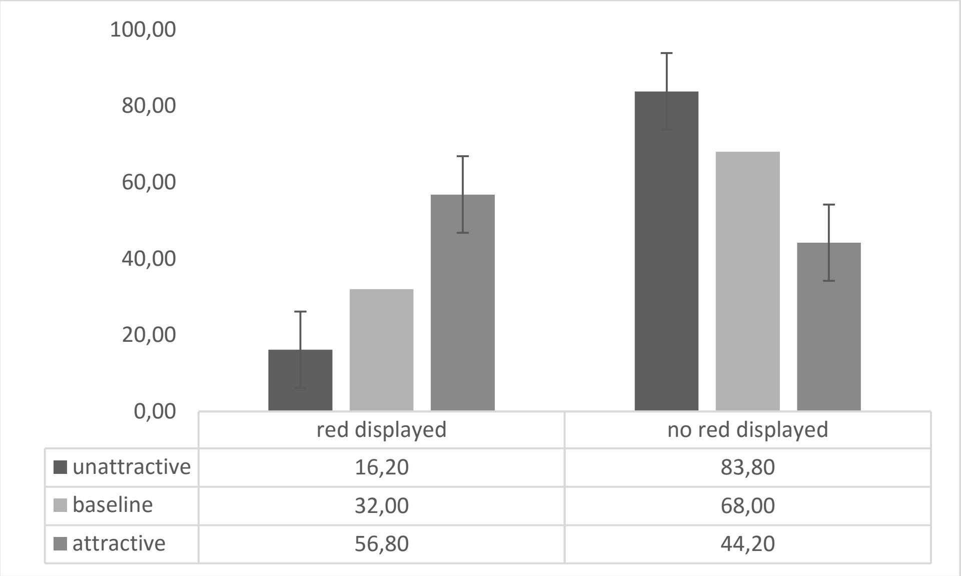

Note. Confidence interval based on a 1%-level of significance
