# Supplementary material for: Strategic Sexual Signals: Women's Display versus Avoidance of the Color Red Depends on the Attractiveness of an Anticipated Interaction Partner
Source: PLoS One. 2016 Mar 9;11(3):e0148501. doi: 10.1371/journal.pone.0148501 (PMC4784733; doi:10.1371/journal.pone.0148501)
Supplement: S1 Table — (PDF) [file pone.0148501.s002.pdf]

Table 1. Red display of women in the unattractive and attractive experimenter condition

**Stimulus attractiveness**	**Red not displayed**	**Red displayed**	**Total**
Attractive	16	21*	37
	34%	77.80%	50%
Unattractive	31	6*	37
	66%	22.20%	50%
Total	47	27	
	100%	100%	

Note. Between columns percentages differ at a .05 level.
